# Post-natal induction of PGC-1α protects against severe muscle dystrophy independently of utrophin

**DOI:** 10.1186/2044-5040-4-2

**Published:** 2014-01-22

**Authors:** Mun Chun Chan, Glenn C Rowe, Srilatha Raghuram, Ian S Patten, Caitlin Farrell, Zolt Arany

**Affiliations:** 1 Cardiovascular Institute, and Center for Vascular Biology Research, Beth Israel Deaconess Medical Center and Harvard Medical School, 330 Brookline Ave, 02215 Boston, MA, USA

**Keywords:** PGC-1α, PGC-1β, mdx, Duchenne Muscle Dystrophy, Utrophin, Neuromuscular junction

## Abstract

**Background:**

Duchenne muscle dystrophy (DMD) afflicts 1 million boys in the US and has few effective treatments. Constitutive transgenic expression of the transcriptional coactivator peroxisome proliferator-activated receptor gamma coactivator (PGC)-1α improves skeletal muscle function in the murine “mdx” model of DMD, but how this occurs, or whether it can occur post-natally, is not known. The leading mechanistic hypotheses for the benefits conferred by PGC-1α include the induction of utrophin, a dystrophin homolog, and/or induction and stabilization of the neuromuscular junction.

**Methods:**

The effects of transgenic overexpression of PGC-1β, a homolog of PGC-1α in mdx mice was examined using different assays of skeletal muscle structure and function. To formally test the hypothesis that PGC-1α confers benefit in mdx mice by induction of utrophin and stabilization of neuromuscular junction, PGC-1α transgenic animals were crossed with the dystrophin utrophin double knock out (mdx/utrn^-/-^) mice, a more severe dystrophic model. Finally, we also examined the effect of post-natal induction of skeletal muscle-specific PGC-1α overexpression on muscle structure and function in mdx mice.

**Results:**

We show here that PGC-1β does not induce utrophin or other neuromuscular genes when transgenically expressed in mouse skeletal muscle. Surprisingly, however, PGC-1β transgenesis protects as efficaciously as PGC-1α against muscle degeneration in dystrophin-deficient (mdx) mice, suggesting that alternate mechanisms of protection exist. When PGC-1α is overexpressed in mdx/utrn^-/-^ mice, we find that PGC-1α dramatically ameliorates muscle damage even in the absence of utrophin. Finally, we also used inducible skeletal muscle-specific PGC-1α overexpression to show that PGC-1α can protect against dystrophy even if activated post-natally, a more plausible therapeutic option.

**Conclusions:**

These data demonstrate that PGC-1α can improve muscle dystrophy post-natally, highlighting its therapeutic potential. The data also show that PGC-1α is equally protective in the more severely affected mdx/utrn^-/-^ mice, which more closely recapitulates the aggressive progression of muscle damage seen in DMD patients. The data also identify PGC-1β as a novel potential target, equally efficacious in protecting against muscle dystrophy. Finally, the data also show that PGC-1α and PGC-1β protect against dystrophy independently of utrophin or of induction of the neuromuscular junction, indicating the existence of other mechanisms.

## Background

Duchenne muscle dystrophy (DMD) is an inexorably fatal recessive X-linked hereditary disease that affects 1 in 3,600 boys in the US and is marked by progressive muscle dystrophy that ultimately leads to paralysis and respiratory and/or cardiac failure [1-5]. The disease is caused by mutations in the dystrophin gene, which encodes for a critical component of the dystroglycan complex (DGC) that connects the extracellular matrix of muscle fibers to the cytoskeleton [4,6,7]. Dysfunctional DGC leads to excess intracellular calcium, mitochondrial dysfunction, loss of sarcolemmal integrity, and ultimately cell death, but the precise pathogenesis of DMD remains unclear [4,6,8]. Therapeutic options for DMD remain limited, and no significant pharmacological advances have been made since the introduction of steroids in the 1970s [1-3]. A deeper understanding of protective mechanisms is needed.

Utrophin is an important component of the neuromuscular junction (NMJ) and bears significant sequence and functional homology to dystrophin [9,10]. Dystrophin-deficient (mdx) mice, in which the dystrophin gene is mutated, are widely used as a model of DMD, and genetic deletion of utrophin superimposed on the mdx background greatly worsens the observed dystrophy, consistent with redundant function between utrophin and dystrophin [10-15]. The dystrophin utrophin double knock-out mouse (mdx/utrn^-/-^) models the severity of human DMD phenotype more faithfully than the parental dystrophin knock-out (mdx) mouse [11,12,15]. Conversely, transgenic expression of utrophin in skeletal muscle significantly improves indices of dystrophy in mdx mice [3,10,16]. Utrophin has thus been proposed as a potential therapeutic target, and efforts are underway to translate these observations to the clinical setting.

Transgenic overexpression in skeletal muscle of peroxisome proliferator-activated receptor gamma coactivator (PGC)-1α has also been shown to markedly improve dystrophy in mdx mice [17]. PGC-1α powerfully regulates broad programs of gene expression by binding to, and co-activating, numerous transcription factors [18-20]. In most tissues, including skeletal muscle, PGC-1α strongly induces mitochondrial biogenesis by co-activating nuclear respiratory factor 1 (NRF1), NRF2, estrogen-related receptor α (ERRα), and other transcription factors. At the same time, PGC-1α induces the expression of a number of ancillary programs, including fatty acid transport and oxidation [21-23]. PGC-1β, a homolog that bears extensive sequence identity with PGC-1α in a number of functional domains, shares many, though not all, of these functions [24-28].

In skeletal muscle, PGC-1α also induces genes that encode components of the neuromuscular junction [17,29]. NRF2, composed of a hetero-tetramer of GA-binding protein α chain (GABPA) and GA-binding protein β chain (GABPB), was in fact independently identified as a key transcription factor in the regulation of NMJ genes. Consistent with this, PGC-1α strongly induces utrophin expression both in cells and in mice that transgenically overexpress PGC-1α in skeletal muscle [17,29,30]. As noted above, these same transgenic mice markedly improve dystrophy in the mdx background [17]. This strong protection has thus widely been ascribed to the induction of utrophin by PGC-1α [17,31-34], although this has never formally been tested. It is also not clear when in development the induction of PGC-1α is most critical, because prenatal changes in dystrophin-deficient muscle means that prenatal expression of PGC-1α may have had important effects [35,36].

Here, we use various transgenic mice to test if induction of utrophin by PGC-1α is necessary for amelioration of muscle damage in DMD models. We find that PGC-1α surprisingly protects against dystrophy independently of utrophin. In fact, PGC-1α efficiently protects the mdx/utrn^-/-^ mice, which, as noted above, model the severity of human DMD more faithfully than the parental mdx mouse [15]. We also show that PGC-1β protects against dystrophy as efficaciously as PGC-1α, even though PGC-1β does not induce utrophin or genes of the NMJ. Finally, we use an inducible transgenic model to show that induction of PGC-1α well after birth can still confer efficient protection against dystrophy, a more plausible therapeutic scenario.

## Methods

### Animals

All animal experiments were performed according to procedures approved by the Beth Israel Deaconess Medical Center’s Institutional Animal Care and Use Committee. C57BL/10ScSn-*Dmd*^mdx^/J mice (mdx mice) [37] and *Utrn*^*tm1Jrs*^*Dmd*^*mdx*^*/J* mice (mdx/utrn^-/-^ mice) [11,13] were obtained from Jackson Laboratories (Bar Harbor, Maine, USA). These mice were crossed with: muscle-specific PGC-1α transgenic mice (MCK-PGC-1α) [38]; muscle-specific PGC-1β transgenic mice (MCK-PGC-1β) [39]; or mice with inducible PGC-1α that possess the muscle specific tetracycline-dependent activator (MCK-tTA) and the PGC-1α coding region under control of the tet-response element promoter (TRE-PGC-1α) transgenic genes (Ind_PGC-1α) [40]. mdx, mdx/MCK-PGC-1α, mdx/MCK-PGC-1β and mdx/Ind_PGC-1α are products of crosses between animals in the C57/BL/6J background (MCK-PGC-1α, MCK-PGC-1β and Ind_PGC-1α mice) and C57/BL/10ScSn (mdx mice) background. All non-dystrophic mice, including wild-type (WT) controls were in the C57/BL/6J background. The mdx/utrn^-/-^ mice as provided by Jackson Laboratories are in a mixed background. Therefore, all experimental groups with these mice were conducted on sibling-mates in mixed background. Sedentary mdx/MCK-PGC-1α, mdx/MCK-PGC-1β and mdx/utrn^-/-^/MCK-PGC-1α mice were analyzed at 5 weeks of age, while exercise experiments were performed on 10-week-old mice (n = 5 per experimental group). mdx/Ind_PGC-1α were induced by removal of doxycycline from chow at 3 weeks of age and experiments performed at 7 weeks of age (n = 5 per experimental group).

### Growth curves and radiograph

Body weight and body length (nose to tail base) of litter mates from mdx/utrn^+/-^ /MCK-PGC-1α male crossed with mdx/utrn^+/-^ females was measured in a blinded fashion from 5 weeks to 15 weeks of age. Radiographs of representative animals in each genotype were taken of anesthetized animals at 13 weeks of age.

### Treadmill exercise

Ten-week-old mice were run for 1 hour or until exhaustion on a treadmill at a 15° downhill angle (1^st^ run). Treadmill speed was steadily increased, starting at 10 m/minute for 5 minutes, then increasing to 15 m/minute for 5 minutes, 20 m/minute for 5 minutes, and finally 25 m/minute for 45 minutes. Blood was drawn 2 hours after the run. This exercise protocol was repeated after a 24 hour resting period (2^nd^ run). Mice were sacrificed 2 hours after the 2^nd^ run.

### Cell culture

Primary satellite cells were isolated and cultured from the entire hind limb of WT mice as previously described [41,42]. Cells were differentiated into myotubes using DMEM with 5% horse serum for 72 hours. Cells were then infected with control (GFP) adenovirus (WT), recombinant PGC-1α adenovirus (Ad-PGC-1α) or recombinant PGC-1β adenovirus (Ad-PGC-1β) at multiplicity of infection of 10 to 30 [43]. Cells were analyzed 48 hours later.

### Evans blue injection and histological analysis

For Evans Blue assay, mice were injected with 1% solution intraperitoneally at a final concentration of 1% volume to body weight 16 hours prior to sacrifice. Tissues were dissected and embedded in OCT compound (VWR, Radnor, Pennsylvania, USA) and flash-frozen. Evans Blue was analyzed by fluorescence microscopy. Sections were also stained with rabbit polyclonal anti-laminin antibody (Abcam, ab11575, Cambridge, Massachusetts, USA) in order to establish fiber boundaries. We used two different staining techniques in this report to calculate centralized nuclei: i) frozen sections were stained with anti-laminin and counterstained with 4',6-diamidino-2-phenylindole (DAPI); ii) tissue was dehydrated and embedded in paraffin and sectioned before hematoxylin and eosin staining. In either case, 20 images were taken at 200× and the number of centralized nuclei to overall number of nuclei counted in a blinded fashion.

### Serum creatine kinase assay

Blood was collected and serum isolated using heparin-coated collection tubes, either by heart puncture or cheek bleed [44,45] Serum Creatine kinase activity was then determined with a Genzyme Creatine Kinase-SL Assay Kit (BioPacific Diagnostic Inc, Vancouver, BC, Canada).

### Real-time polymerase chain reaction

Total RNA was isolated from mouse tissue using the TRIzol (Invitrogen, Grand Island, NY, USA) reagent, while RNA from cultured cells was isolated using the Turbocapture (Qiagen, Valencia, California, USA) method. Samples for real-time PCR analyses were reverse transcribed (Applied Biosystems, Foster City, California, USA), and quantitative real-time PCRs (qPCRs) were performed on the cDNAs in the presence of fluorescent dye (SYBR green) (Bio-Rad, Hercules, California, USA). Relative expression levels were determined using the comparative cycle threshold method [46].

### Western blotting

Protein from quadriceps (100 μg) was run on a 4% polyacrylamide gel, transferred to nitrocellulose membrane, and blotted for utrophin using an anti-utrophin antibody (H-300, Santa Cruz Biotech, Santa Cruz, California USA). As a control, equivalent protein was run on a 10% polyacrylamide gel, transferred and probed with anti-pan-actin antibody (Cell Signaling Technology Inc, Boston, Massachusetts, USA),

### Statistical analysis

The data are presented as means ± SEM. Statistical analysis was performed with Student’s t-test for all *in vitro* experiments, analysis of variance for all *in vivo* experiments, and Log-rank (Mantel-Cox) test for the Kaplan–Meier curves. *P* values <0.05 were considered statistically significant.

## Results

### PGC-1β, in contrast to PGC-1α, does not induce expression of utrophin and other neuromuscular junction genes

**Figure 1:**
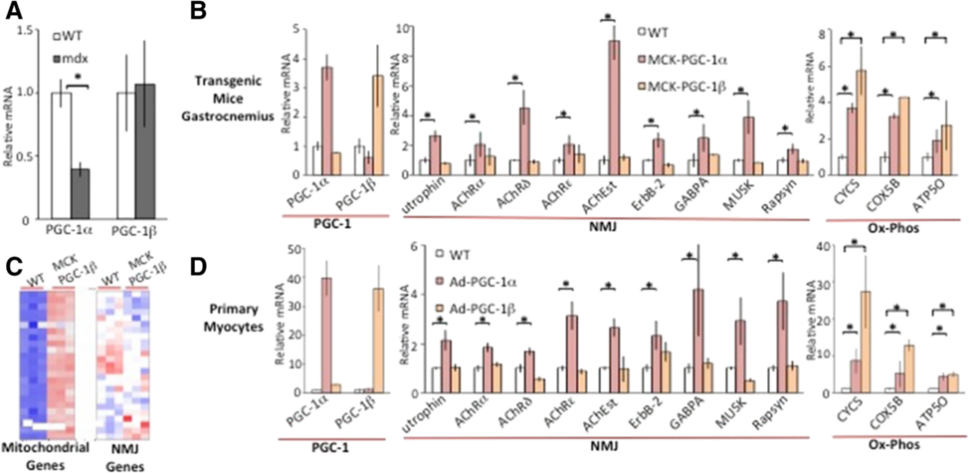
**Peroxisome proliferator-activated receptor gamma coactivator (PGC)-1β, in contrast to PGC-1α, does not induce expression of utrophin and other neuromuscular junction (NMJ) genes. (A)** Expression of PGC-1α and PGC-1β in the gastrocnemius of 5-week-old male control (wild-type; WT) and dystrophin-deficient (mdx) mice was examined by isolating mRNA, performing reverse transcription, followed by quantitative PCR (n = 4). **(B)** Five-week-old male mice with transgenic overexpression of PGC-1α in muscle cells (MCK-PGC-1α), or male mice with transgenic overexpression of PGC-1β in muscle cells (MCK-PGC-1β) (n = 5 each group). Genes of the NMJ and genes involved in oxidative phosphorylation (Ox-Phos) were analyzed. Bars depict relative mRNA expression. **(C)** Heat map of the 25 most highly expressed mitochondrial genes (left) and the 25 most highly expressed NMJ genes (right) from microarrays of RNA isolated from quadriceps of WT and MCK-PGC-1β mice. **(D)** Primary myotubes in cell culture were infected with control adenovirus (WT), recombinant PGC-1α adenovirus (Ad-PGC-1α) or recombinant PGC-1β adenovirus (Ad-PGC-1β). Cells were harvested 48 hours after infection and mRNA expression determined by quantitative PCR. Bars depict relative mRNA expression, error bars represent SEM. *P < 0.05. AChR, acetylcholine receptor.

We began our studies by investigating the role of PGC-1β, if any, in the pathogenesis of, and possible protection against, muscle dystrophy. Increased PGC-1α has been shown to ameliorate muscle damage in mdx mice [17,31-34]. PGC-1α has also been shown to be decreased in a dog model of DMD [47], suggesting that loss of PGC-1α protection may contribute to the dystrophy. Similarly, we found that PGC-1α mRNA expression, measured by qPCR, is significantly reduced in gastrocnemius muscle from 5-week-old mdx mice (Figure 1A). In contrast, expression of PGC-1β was not decreased in mdx mice, suggesting that PGC-1β may play a different role than PGC-1α in mdx pathogenesis.

Because the anti-dystrophic effects of PGC-1α have widely been ascribed to induction of the NMJ, and utrophin in particular, we next tested if PGC-1β also regulates this program. Five-week-old male mice with transgenic overexpression of PGC-1α in muscle cells (MCK-PGC-1α) showed significant increase in expression of genes involved in the NMJ (for example, utrophin, acetylcholine receptor (AChR)α, AChRδ, AChRϵ, acetylcholine esterase (AChEst), ErbB-2, GABPA, MUSK and Rapsyn) in the gastrocnemius, compared to littermate control animals (WT) (Figure 1B). Unlike PGC-1α, however, equivalent transgenic increase in PGC-1β expression in muscle cells (MCK-PGC-1β) did not increase expression of genes of the NMJ (Figure 1B). In contrast, genes associated with oxidative phosphorylation (ox-phos) were significantly induced by transgenic overexpression of both PGC-1α and PGC-1β, as published previously [24]. Induction of ox-phos genes was equivalent if not slightly higher in MCK-PGC-1β animals, demonstrating that the non-induction of genes of the NMJ cannot be explained by poor activity of the transgenic PGC-1β. Unbiased microarray analyses of RNAs from the quadriceps of MCK-PGC-1β mice versus littermate controls similarly revealed increased expression of mitochondrial genes compared to WT, but no change in genes of the NMJ (Figure 1C). Similarly, in cultured differentiated primary myotubes, infection with recombinant PGC-1α adenovirus (Ad-PGC-1α) increased expression of both NMJ genes and ox-phos genes, while infection with recombinant PGC-1β (Ad-PGC-1β) induced only ox-phos genes and had no effect on NMJ genes (Figure 1D). Together, these results indicate that, unlike PGC-1α, PGC-1β does not regulate expression of NMJ genes or utrophin in muscle cells or *in vivo*.

### PGC-1β prevents damage in dystrophic muscle

**Figure 2:**
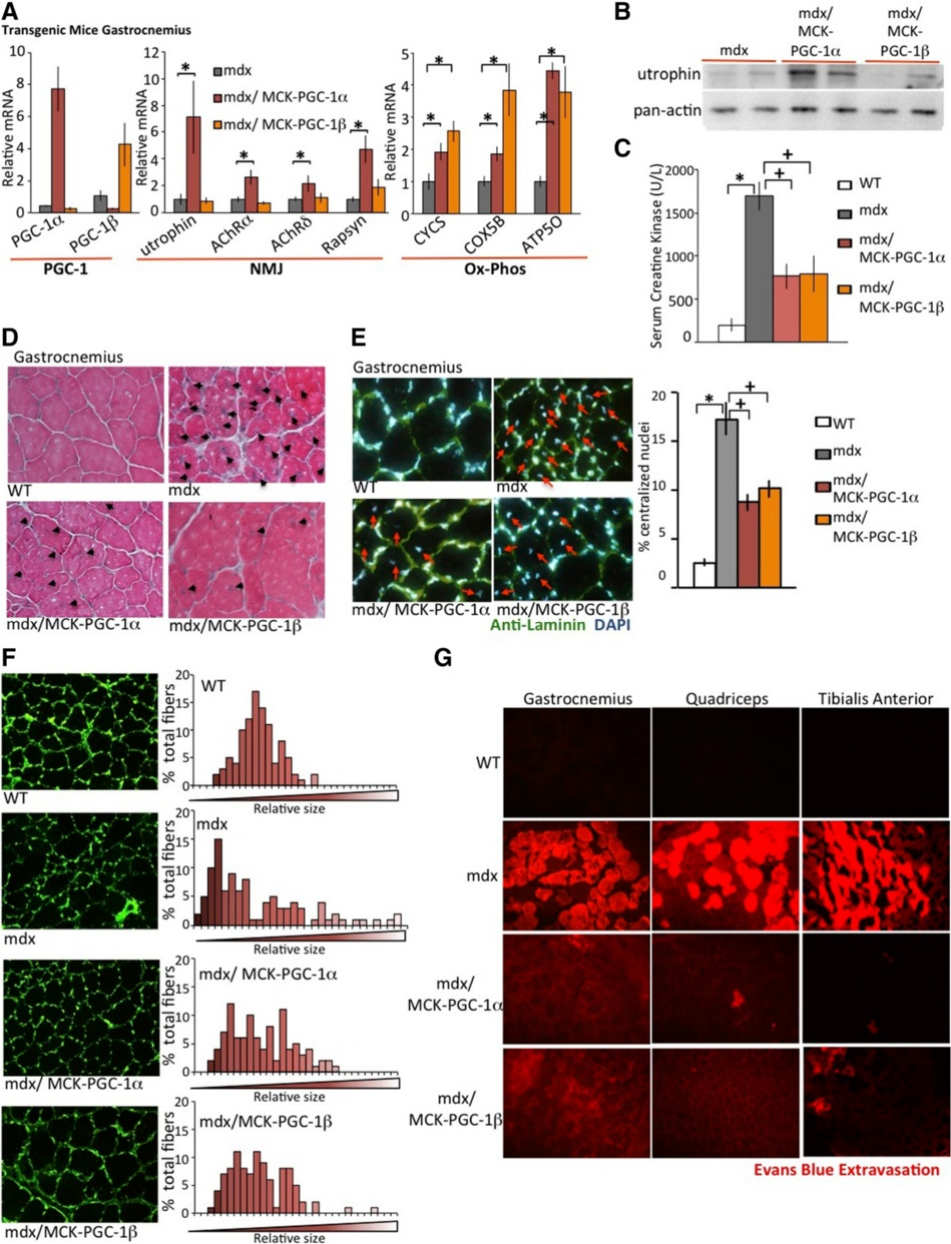
**Peroxisome proliferator-activated receptor gamma coactivator (PGC)-1β ameliorates muscle damage in dystrophin-deficient (mdx) mice without induction of utrophin or other neuromuscular junction (NMJ) genes. (A)** Five week old control (WT), mdx, mdx mice with overexpression of PGC-1α in muscle cells (mdx/MCK-PGC-1α), or mdx mice with overexpression of PGC-1β in muscle cells (mdx/MCK-PGC-1β) (n = 5 each group) were sacked, and quantitative PCR was performed on mRNA isolated from the gastrocnemius. Genes of the NMJ and genes involved in oxidative phosphorylation (Ox-Phos) were analyzed. **(B)** Quadriceps from mdx, mdx/MCK-PGC-1α and mdx/MCK-PGC-1β were harvested and subjected to immunoblot analysis with anti-utrophin antibody and anti-pan-actin antibody. Results are representative of three independent experiments. **(C)** Five-week-old WT, mdx, MCK-PGC-1α and MCK-PGC-1β mice were sacked and serum creatine kinase activity was measured. Bars depict average serum creatine kinase activity (n = 5). **(D)** Histological sections from gastrocnemius of 5-week-old WT, mdx, MCK-PGC-1α and MCK-PGC-1β mice were prepared and stained with hematoxylin and eosin. Black arrows indicate centralized nuclei. **(E)** Histological sections from gastrocnemius of 5-week-old WT, mdx, MCK-PGC-1α and MCK-PGC-1β mice were stained with anti-laminin (green) to show muscle fiber boundaries and 4',6-diamidino-2-phenylindole (DAPI) to stain nuclei (blue). Representative images shown (left), with red arrows indicating centralized nuclei. Bars (right) depict percentage of nuclei counted which were centralized (n = 5). **(F)** Images of the same section were analyzed for relative fiber size using ImageJ. Bar histograms represent size distribution of the muscle fibers (right). **(G)** Five-week-old WT, mdx, MCK-PGC-1α and MCK-PGC-1β mice were injected intraperitoneally with 1% v/w Evans Blue solution and sacrificed 16 hours later. Histological sections from gastrocnemius, quadriceps and tibialis anterior were analyzed for dye incorporation into muscle fibers by fluorescence microscopy. Representative images are presented. Error bars represent SEM. *P < 0.05. AChR, acetylcholine receptor.

The prevailing model of the mechanism by which PGC-1α ameliorates muscle damage is by positively regulating expression of genes of the NMJ, including utrophin. The above results demonstrating that PGC-1β does not induce this program thus suggest that PGC-1β transgenesis would fail to recapitulate the protection against dystrophy seen in PGC-1α transgenic animals. To test this notion, we crossed mdx animals with MCK-PGC-1β (and, as positive controls, MCK-PGC-1α) animals. Transgenic expression of PGC-1α in mdx mice (mdx/MCK-PGC-1α) increased mRNA expression of NMJ genes and utrophin, as published previously (Figure 2A). Transgenic expression of PGC-1β (mdx/MCK-PGC1β) did not induce this program, echoing the findings in non-dystrophic mice (Figure 1). Both PGC-1α and PGC-1β induced the expression of ox-phos genes (Figure 2A). Utrophin protein was induced in mdx/MCK-PGC-1α quadriceps compared to quadriceps from mdx mice alone (Figure 2B), while mdx/MCK-PGC-1β mice showed no significant increase in utrophin protein. Therefore, unlike PGC-1α, PGC-1β does not induce utrophin or other proteins of the NMJ in mdx mice.

Next, we performed various experiments to determine the relative amount of damage and function in mdx, mdx/MCK-PGC-1α, and mdx/MCK-PGC-1β mice. We first tested the level of serum creatine kinase. There was significant increase in free creatine kinase in the serum of mdx compared to WT mice (Figure 2C), consistent with increased muscle damage. The amount of serum creatine kinase was significantly suppressed in mdx/MCK-PGC-1α compared to mdx alone, as previously shown [17], consistent with amelioration of dystrophy by PGC-1α expression. Surprisingly, mdx/MCK-PGC-1β also had significantly lower levels of serum creatine kinase compared to mdx alone, suggesting that PGC-1β is also protective in the mdx model.

We next stained cross-sections of the gastrocnemius with hematoxylin and eosin (Figure 2D). We observed that mdx mice had a greater number of centralized nuclei and smaller fiber size as previously reported [48]. Increased transgenic expression of PGC-1α appeared to improve these phenotypes (Figure 2D). In order to better visualize and quantify these differences, we stained cross-sections of the gastrocnemius with anti-laminin to stain boundary of muscle fibers and counterstained with DAPI. Consistent with previous studies, mdx mice had a larger variation in fiber size and a larger proportion of small fibers compared to WT mice [48] (Figure 2E). The muscle fibers in mdx/MCK-PGC-1α had a lower variation in size, and had fewer small fibers compared to mdx alone. Muscle fibers in the mdx/MCK-PGC-1β were similar to mdx/MCK-PGC-1α. PGC-1β transgenesis thus partly rescued the fiber size defect seen in mdx mice. We next measured the percentage of nuclei found in the center of muscle fibers (Figure 2F), an established model of estimating the number of post-damage regenerating fibers. The number of centralized nuclei in gastrocnemius of the mdx mice was approximately 17%, while essentially no central nuclei were seen in WT animals. mdx/MCK-PGC-1α mice had approximately 50% decrease in the number of centralized nuclei, consistent with partial rescue of the mdx phenotype. The number of centralized nuclei in mdx/MCK-PGC-1β mice was similarly reduced compared to mdx alone. Similar findings were seen in quadriceps and tibialis anterior (data not shown).

The extravasation of Evans Blue dye into the muscle of mice is a standard measure of vascular and sarcolemmal integrity. As shown in Figure 2G, there was a significant increase in the amount of Evans Blue leakage into the gastrocnemius, the quadriceps and the tibialis anterior of mdx mice compared to WT, consistent with muscle damage and necrosis in mdx mice. Increased expression of PGC-1α in mdx/MCK-PGC-1α mice lowered the intensity of Evans Blue dye in the different muscles, as previously reported [17]. Surprisingly again, increased expression of PGC-1β in mdx/MCK-PGC-1β reduced Evans Blue extravasation equally effectively (Figure 2G).

### PGC-1β improves exercise capacity of dystrophic mice

**Figure 3:**
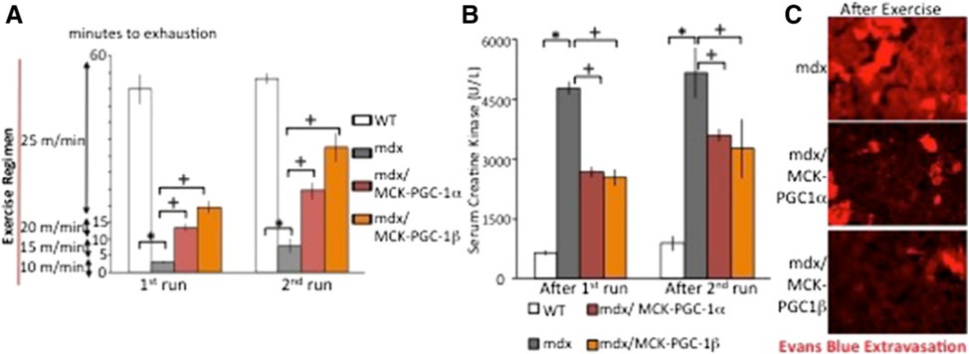
**Peroxisome proliferator-activated receptor gamma coactivator (PGC)-1α and PGC-1β improve muscle function in exercisingdystrophin-deficient (mdx) mice. (A)** Ten-week-old control (wild-type; WT), mdx, mdx mice with transgenic overexpression of PGC-1α in muscle cells (mdx/MCK-PGC-1α), or mdx mice with transgenic over-expression of PGC-1β in muscle cells (mdx/MCK-PGC-1β) (n = 5 each group) were run for 1 hour or until exhaustion on a treadmill at a 15° downhill angle with steadily increasing speed as described in the methods (1^st^ run). This exercise protocol was repeated after a 24 hour resting period (2^nd^ run). Bars depict average running time. **(B)** Two hours after the first run, blood was drawn from mice by submandibular bleeding. Serum creatine kinase was determined (1^st^ run). Two hours after the second run, mice were sacked and blood was drawn. Serum creatine kinase was determined (2^nd^ run). **(C)** Evans Blue was injected intraperitoneally 16 hours before sacking and histological sections from gastrocnemius were analyzed by fluorescence microscopy. Error bars represent SEM. *P < 0.05.

We next tested the effect of PGC-1α or PGC-1β on mdx mice subjected to eccentric physical exercise:- that is, downhill running. The muscle damage observed in mdx mice subjected to eccentric exercise more closely resembles muscle damage seen in DMD patients [49]. WT, mdx, mdx/MCK-PGC-1α and mdx/MCK-PGC-1β mice were made to run on a treadmill for 1 hour at an decline of 15° downhill. This exercise regimen was repeated 24 hours after the first run. WT mice were able to complete this exercise regimen, but mdx mice were exhausted within the first 5 to 10 minutes of the regimen (Figure 3A), as shown previously [17]. Both mdx/MCK-PGC-1α and mdx/MCK-PGC-1β mice were able to run for significantly longer compared to mdx alone on both the 1^st^ and 2^nd^ run (Figure 3A). Serum was drawn from the animals 2 hours after each run, and serum creatine kinase measured. There was significant increase in creatine kinase of mdx mice compared to WT mice, consistent with significant induction of muscle damage by eccentric exercise (Figure 3B). Compared to mdx alone, both mdx/MCK-PGC-1α and mdx/MCK-PGC-1β had significantly lower levels of serum creatine kinase, consistent with lower levels of muscle damage (Figure 3B). In fact, the improvement in creatine kinase leak seen in MCK-PGC-1 mice is likely an underestimate of true improvements conferred by PGC-1 transgenesis, because the transgenic mice were able to run longer than the mdx controls. Similarly, muscle damage as determined by Evans Blue leakage into the muscle fibers was lower in the mdx/MCK-PGC-1α and mdx/MCK-PGC-1β compared to mdx alone (Figure 3C). Together, these results indicate that overexpression of PGC-1α or PGC-1β prevents muscle damage in mdx mice in response to eccentric physical exercise.

Taken together, these data show that PGC-1β is as effective as PGC-1α in ameliorating muscle damage in both sedentary and eccentrically exercised mdx mice. As described above, however, overexpression of PGC-1β in myotubes or *in vivo* does not induce utrophin or other components of the NMJ. This observation strongly suggests that the mechanism by which PGC-1α and PGC-1β ameliorates muscle damage in mdx mice is not related to induction of components of the NMJ.

### Increased PGC-1α expression in dystrophic muscle ameliorates damage in the absence of utrophin

**Figure 4:**
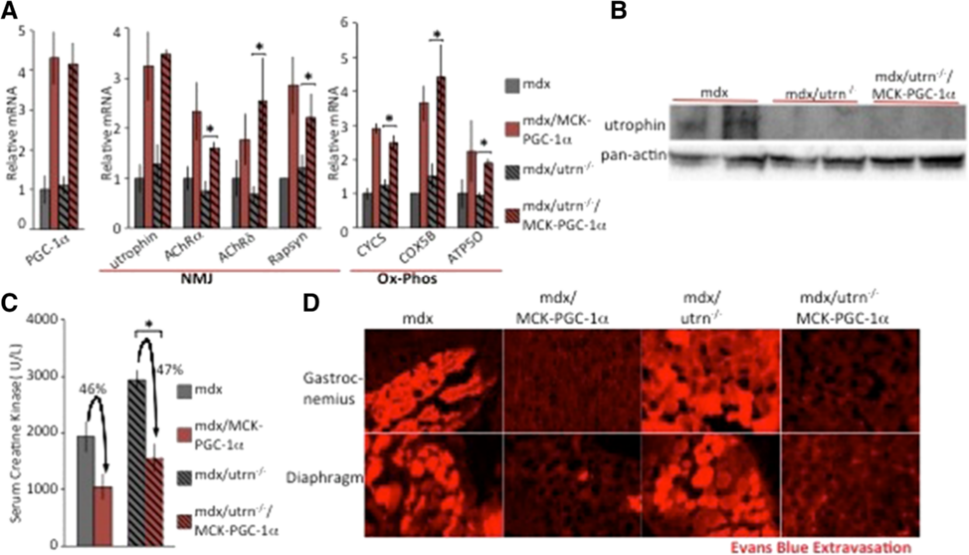
**Peroxisome proliferator-activated receptor gamma coactivator (PGC)-1α ameliorates muscle damage in dystrophin/utrophin-deficient mice. (A)** Five-week-old dystrophin-deficient male mice (mdx), PGC-1α muscle-specific transgenic in an mdx background (mdx/MCK-PGC-1α), dystrophin/utrophin-deficient mice (mdx/utrn^-/-^), and PGC-1α muscle-specific transgenic in dystrophin/utrophin-deficient mice (mdx/utrn^-/-^/MCK-PGC-1α) were sacrificed and quantitative PCR performed on gastrocnemius (n = 5 per group). Bars depict relative mRNA expression, error bars represent SEM. **(B)** Quadriceps from 5-week-old mdx, mdx/utrn^-/-^ and mdx/utrn^-/-^/MCK-PGC-1α were harvested and subjected to immunoblot analysis with anti-utrophin antibody and anti-pan-actin antibody as loading control. Results are representative of three independent experiments. **(C)** Five-week-old male mdx, mdx/MCK-PGC-1α, mdx/utrn^-/-^ and mdx/utrn^-/-^/MCK-PGC-1α mice were sacked and blood was drawn. Serum creatine kinase was measured. Bars represent average level of serum creatine kinase. Error bars represent SEM (n = 5). **(D)** Evans Blue was injected intraperitoneally 16 hours before sacking into 5-week-old male mdx, mdx/MCK-PGC-1α, mdx/utrn^-/-^ and mdx/utrn^-/-^/MCK-PGC-1α. Histological sections from gastrocnemius and diaphragm were analyzed by fluorescence microscopy. *P < 0.05. AChR, acetylcholine receptor.

As outlined above, our results with MCK-PGC-1β mice support the hypothesis that the beneficial impact of PGC-1α in mdx mice occurs independently of utrophin induction. We next directly tested this hypothesis by using mice that lack both dystrophin and utrophin (mdx/utrn^-/-^). Previous reports have shown that these mice suffer from a more severe dystrophy compared to mdx mice alone [11-13]. The progression of dystrophy and reduced lifespan of these mice more closely recapitulates progression of DMD in humans [15]. We first tested the effect of overexpression of PGC-1α in muscle of these mice (mdx/utrn^-/-^/MCK-PGC-1α) on induction of NMJ and ox-phos genes. Similar to mdx mice, components of NMJ and ox-phos genes were induced in the mdx/utrn^-/-^ mice (Figure 4A). The mutation in the *utrn* gene results in premature translation termination, and utrophin mRNA is still present (as previously reported [11]) and induced by PGC-1α (Figure 4A). However, utrophin protein is absent in mdx/utrn^-/-^ and mdx/utrn^-/-^/MCK-PGC-1α mice (Figure 4B), as expected.

We next tested the effects of PGC-1α expression on muscle damage in mdx/utrn^-/-^ mice. Serum creatine kinase levels were higher in mdx/utrn^-/-^ mice compared to mdx alone, consistent with a more severe progression of dystrophy (Figure 4C). PGC-1α expression reduced serum creatine kinase by 47%, comparing mdx/utrn^-/-^/MCK-PGC-1α mice to mdx/utrn^-/-^ alone. This is similar to the 46% reduction in serum creatine kinase with expression of PGC-1α in mdx mice (Figure 4C), demonstrating that PGC-1α protects against muscle dystrophy as efficiently in the absence of utrophin as in its presence. Similar results were observed with Evans Blue incorporation experiments. Evans Blue staining in the fibers of the gastrocnemius and diaphragm was higher in mdx/utrn^-/-^ mice compared to mdx mice (Figure 4D), and the intensity of staining was markedly reduced in mdx/utrn^-/-^/MCK-PGC-1α compared to mdx/utrn^-/-^ alone (Figure 4D).

**Figure 5:**
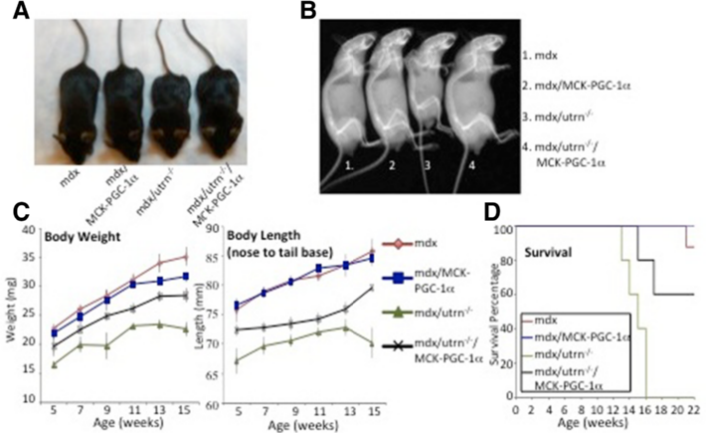
**Peroxisome proliferator-activated receptor gamma coactivator (PGC)-1α reverses poor growth and kyphosis in dystrophin/utrophin-deficient mice. (A)** Photograph of representative 5-week-old dystrophin deficient male mice (mdx), PGC-1α muscle-specific transgenic in an mdx background (mdx/MCK-PGC-1α), dystrophin/utrophin-deficient mice (mdx/utrn^-/-^), and PGC-1α muscle-specific transgenic in dystrophin/utrophin-deficient mice (mdx/utrn^-/-^/MCK-PGC-1α) indicating difference in size and pronounced kyphosis in mdx/utrn^-/-^ that is largely reversed in mdx/utrn^-/-^/MCK-PGC-1α animals. **(B)** Representative radiograph of 13-week-old male mdx, mdx/MCK-PGC-1α, mdx/utrn^-/-^ and mdx/utrn^-/-^/MCK-PGC-1α mice showing small size and pronounced kyphosis in mdx/utrn^-/-^ mice is largely reversed in mdx/utrn^-/-^/MCK-PGC-1α mice. **(C)** Body weight (left) and body length from nose to tail base (right) of mdx (n = 8), mdx/MCK-PGC-1α (n = 8), mdx/utrn^-/-^ (n = 5) and mdx/utrn^-/-^/MCK-PGC-1α (n = 5) male mice were measured every 2 weeks from 5 to 15 weeks of age. Graph represents average body weight and average body length, error bars SEM. **(D)** Kaplan-Meier survival plot of mdx (n = 8), mdx/MCK-PGC-1α (n = 8), mdx/utrn^-/-^ (n = 5) and mdx/utrn^-/-^/MCK-PGC-1α (n = 5) male mice with time. *P* < 0.02 by Log-rank (Mantel-Cox) test for mdx/utrn^-/-^ compared to mdx/utrn^-/-^/MCK-PGC-1α cohorts.

The mdx/utrn^-/-^ mice suffer from poor growth, significant kyphosis and reduced life-span compared to mdx mice [12,13]. We examined if overexpression of PGC-1α in skeletal muscle would affect any of these phenotypes. Physical examination of 5-week-old mice revealed that the mdx/utrn^-/-^ mice were smaller and appeared more hunched compared to mdx mice. In comparison, mdx/utrn^-/-^/MCK-PGC-1α mice appeared larger than mdx/utrn^-/-^ mice (Figure 5A) and appeared indistinguishable from mdx mice. Radiography of mdx/utrn^-/-^ revealed severe kyphosis compared to mdx mice (Figure 5B). In contrast, kyphosis in mdx/utrn^-/-^/MCK-PGC-1α was largely absent (Figure 5B). Body weight and body length (nose to tail) of mdx, mdx/MCK-PGC-1α, mdx/utrn^-/-^, and mdx/utrn^-/-^/MCK-PGC-1α mice were measured between 5 and 15 weeks of age (Figure 5C). There was no significant difference in body weight or length between mdx and mdx/MCK-PGC-1α mice. mdx/utrn^-/-^, however, were significantly lighter and smaller compared to mdx mice alone (Figure 5C). In contrast, mdx/utrn^-/-^/MCK-PGC-1α mice were heavier, and had longer body length compared to mdx/utrn^-/-^ mice. The life span of mdx mice is reported to be up to 2 years. The life span of the mdx/utrn^-/-^ mice, in contrast, is shortened, with all mice in our cohort dying at between 13 and 17 weeks of age (Figure 5D). The mdx/utrn^-/-^/MCK-PGC-1α mice showed an increase in life span, with 60% of mice remaining alive after 18 weeks (compared to 0% of mdx/utrn^-/-^ mice) (*P* < 0.02 versus mdx/utrn^-/-^ mice).

Together, these results demonstrate that overexpression of PGC-1α in muscle rescues dystrophy independently of utrophin. The results also show that muscle PGC-1α dramatically improves myopathy and whole-body sequelae in mdx/utrn^-/-^ mice, a more severe dystrophic model that more closely approximates the human disease.

### Postnatal increase in PGC-1α expression in muscle improves dystrophic muscle

**Figure 6:**
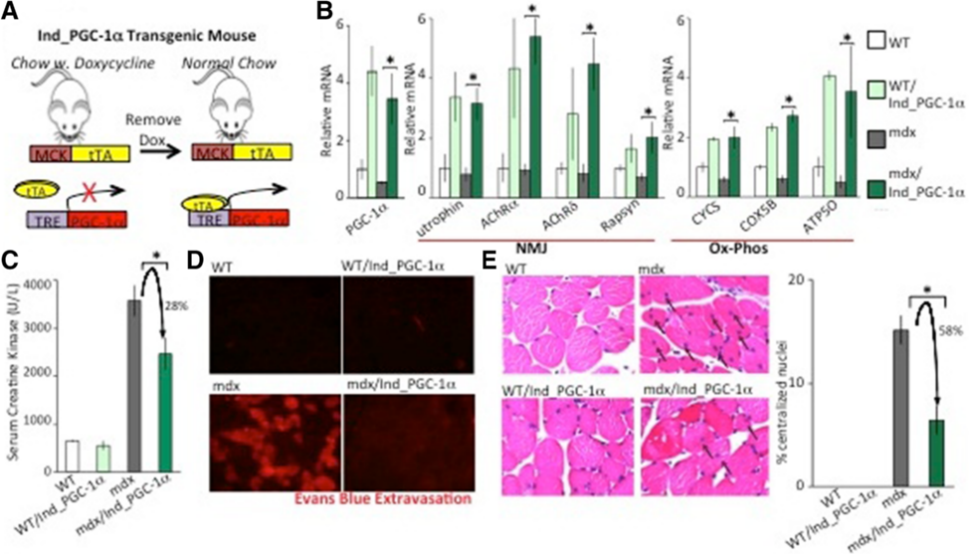
**Post-natal expression of peroxisome proliferator-activated receptor gamma coactivator (PGC)-1α ameliorates muscle damage in dystrophin-deficient (mdx) mice. (A)** The inducible PGC-1α mouse (Ind_PGC-1α) possess two transgenic genes: the muscle specific tetracycline-dependent activator (MCK-tTA); and PGC-1α coding region under control of the tet-response element promoter (TRE_PGC-1α). Removal of doxycycline (dox) allows tTA to bind the TRE region and increase expression of PGC-1α. Mice were kept off dox for 4 weeks (from 3 to 7 weeks of age) prior to the experiment. **(B)** Nine-week-old male control (wild-type; WT), inducible PGC-1α muscle-specific transgenic mice (WT/Ind_PGC-1α), mdx, and inducible PGC-1α muscle-specific transgenic mice in an mdx background (mdx/Ind_PGC-1α) were sacked and quantitative PCR performed on mRNA from gastrocnemius (n = 5 per group). Bars depict relative mRNA expression. **(C)** Seven-week-old WT, WT/Ind_PGC-1α, mdx and mdx/Ind_PGC-1α mice were sacked and blood was drawn. Serum creatine kinase was measured. Bars represent average level of serum creatine kinase. **(D)** Evans Blue was injected intraperitoneally 16 hours before sacking into 7-week-old old WT, WT/Ind_PGC-1α, mdx and mdx/Ind_PGC-1α mice. Histological sections from gastrocnemius were analyzed by fluorescence microscopy. **(E)** Histological sections from quadriceps of 7-week-old WT, WT/Ind_PGC-1α, mdx and mdx/Ind_PGC-1α mice were stained with hematoxylin and eosin. The number of nuclei found in the center of the cell as opposed to on the periphery was counted. Representative images shown (left) with small black arrows indicating centralized nuclei. Bars (right) depict percentage of nuclei counted which were centralized (n = 5). Error bars represent SEM. *P < 0.05. AChR, acetylcholine receptor; NMJ, neuromuscular junction; Ox-phos, oxidative phosphorylation.

The above experiments underscore PGC-1α as a potential therapeutic target for DMD. However, in all of these mice as well as those previously published [17], PGC-1α overexpression in skeletal muscle begins *in utero*. Dystrophin-deficient muscle reveals significant abnormalities even prenatally [35,36], suggesting that prenatal expression of PGC-1α may have had important beneficial effects that would be difficult to recapitulate in the clinical setting. We thus wanted to test if post-natal increase in PGC-1α expression can reduce muscle damage in mdx mice. To conduct this experiment, we used double-transgenic mice (MCK-tTA/TRE-PGC-1α) as described in Figure 6A and the Methods section. Induction of PGC-1α thus occurs only with removal of doxycycline from chow (Figure 6A). These mice were then additionally bred into the mdx background. Mice were switched to chow lacking doxycycline at the age of 3 weeks and were examined 4 weeks later. We first confirmed that induction of PGC-1α was successful, observing an approximate 4-fold increase in PGC-1α expression, both in mdx and WT backgrounds (Figure 6B), and mildly less than observed in constitutive mdx/MCK-PGC-1α mice (Figure 2A). We also observed corresponding increase in NMJ and ox-phos genes with PGC-1α induction in both genetic backgrounds (Figure 6B).

We next tested if induction of post-natal induction of PGC-1α affects dystrophic muscle damage in mdx mice. Serum creatine kinase was significantly reduced by 28% (Figure 6C) after PGC-1α induction. Evans Blue staining of muscle was also reduced in mdx/Ind_PGC-1α mice compared to mdx mice (Figure 6D). Lastly, we examined the number of central nuclei found within the muscle fibers, an established measure of post-damage regenerating fibers in mice. There were no centralized nuclei observed in WT or WT/Ind_PGC-1α mice, while approximately 18% of nuclei in mdx mice were found in the center of cells (Figure 6E). Induction of PGC-1α significantly decreased the number of centralized nuclei by 58% compared to mdx mice. From these results, we conclude that post-natal induction of PGC-1α is able to efficiently prevent muscle damage in mdx mice.

## Discussion

We demonstrate in this study that: both PGC-1α and PGC-1β improve muscle function in dystrophic mouse models; that they do so independently of utrophin induction; that PGC-1α improves muscle function even if delivered only post-natally; and that even the more severe mdx/utrn^-/-^ model of DMD is efficiently rescued by PGC-1α.

The induction of utrophin efficiently rescues the absence of dystrophin in muscle [2,3,10,16,50] and has thus generally been assumed to explain the benefits conferred by PGC-1α [17,31,33]. The present study, however, demonstrates that this is not the case. The data demonstrate the existence of other beneficial pathways. Identifying these pathways will be of great interest, especially in light of the efficient protection afforded by PGC-1α induction. A number of potential mechanisms exist. ATP deficiency may contribute to myocyte death, and PGC-1 induction, leading to mitochondrial biogenesis and increased OXPHOS capacity, significantly increases the capacity for ATP generation. Mitochondria also provide a large buffer for cytosolic calcium, and cytosolic calcium overload in dystrophic muscle is thought to contribute to the activation of calpains and caspases, ultimately leading to myocyte apoptosis. PGC-1-induced mitochondrial biogenesis may thus buffer cytosolic calcium and help prevent these events. PGC-1s have also been shown widely, especially in neurobiological contexts, to reduce cellular levels of reactive oxygen species (ROS) [51-53], and ROS have also been implicated in dystrophic pathophysiology [54-57]. Finally, PGC-1α may suppress the susceptibility of mitochondrial permeability transition pore (PTP) to open [58], again minimizing the tendency to apoptosis. The fact that one or more of these mechanisms must underlie the powerful PGC-1α-mediated protection underscores the need to better understand these processes.

Despite the fact that it does not induce genes of the NMJ, our data supports the use of PGC-1β as a possible alternative therapeutic target in DMD. Why PGC-1β fails to induce utrophin and other NMJ genes is not clear. PGC-1β likely interacts efficiently with NRF2, because it strongly induces mitochondrial genes [19,24]. The specific inability to induce genes of the NMJ thus likely does not stem from an inability to bind GABPA/B tetramers (NRF2). Post-translational modification of GABPA and PGC-1α by p38MAPK has been shown to be important for induction of NMJ genes [17], and this process may differ with PGC-1β. The p38MAPK sites in PGC-1α are, for example, not conserved in PGC-1β.

PGC-1α and β strongly drive the formation of oxidative fibers at the expense of glycolytic fibers [38-40]. DMD in humans has been shown to affect type II glycolytic fibers more severely than type I oxidative fibers [59]. This observation has been attributed to the observation that utrophin expression is higher in oxidative fibers, and that utrophin protein in these fibers can be found extra-synaptically, where it would more easily compensate for loss of dystrophin [32,60]. Our current work would suggest, however, that utrophin expression and distribution does not underlie these differences in myofiber susceptibility to DMD, underscoring the need to better understand other protective mechanisms induced by the PGC-1s.

Dystrophy in mdx/utrn^-/-^ mice is much more severe than in mdx mice alone. The mice undergo significant muscle degeneration early in life, with pronounced systemic consequences such as kyphosis, delayed growth, and eventual premature death by 3 to 4 months of age [11-14]. mdx mice, on the other hand, recover efficiently from their repeated bouts of dystrophy, do not have overt systemic phenotypes, and have a normal life span [15,61]. The mdx/utrn^-/-^ mouse model thus recapitulates the clinical spectrum of human DMD much more faithfully than do mdx mice. Our data showing that PGC-1α transgenesis efficiently rescues all of these phenotypes in mdx/utrn^-/-^ mice thus underscores the potential clinical value of targeting PGC-1α and/or its mechanism(s) of action. It will also be of interest to test if PGC-1α can rescue other more severe models of dystrophy, such as telomerase-deficient mice [62].

Together, these results strongly support PGC-1α as a potential therapeutic target for DMD. One approach to this target is gene therapy. Recent reports have suggested that viral or plasmid delivery of PGC-1α post-natally can improve muscle function in mdx mice [32,63]. Our data support this conclusion, and show that PGC-1α expression specifically in myotubes likely mediates this protection. Another approach could use pharmaceutical activation of the PGC-1α pathway. Recent reports have reported benefits in mdx mice with administration of activators of AMPK, SIRT1, or PPARδ, all of which also activate PGC-1α [34,64-66].

## Conclusions

Our data demonstrate that increased expression of either PGC-1α or PGC-1β in skeletal muscle ameliorates muscle damage in mouse models of DMD. We show that this benefit occurs independently of utrophin or increases in NMJ components. We also demonstrate that post-natal induction of PGC-1α is also beneficial in mdx mice, and that even the more severe mdx/utrn^-/-^ model of DMD is efficiently rescued by PGC-1α. PGC-1α and PGC-1β thus represent viable therapeutic targets for DMD, but the mechanisms by which they reduce muscle damage in mouse models of DMD remain unclear and should be the subject of future investigations.

## Abbreviations

AChR: acetylcholine receptor; AChEst: acetylcholine esterase; DGC: dystroglycan complex; DMD: Duchenne muscle dystrophy; DMEM: Dulbecco's modified Eagle's medium; ERRα: estrogen-related receptor α; GABPA: GA-binding protein α chain; GABPB: GA-binding protein β chain; mdx: dystrophin-deficient; NMJ: neuromuscular junction; NRF: nuclear respiratory factor; ox-phos: oxidative phosphorylation; PCR: polymerase chain reaction; PGC: peroxisome proliferator-activated receptor gamma coactivator; qPCR: quantitative real-time PCR; ROS: reactive oxygen species; WT: wild-type.

## Competing interests

The authors declare that they have no competing interests.

## Authors’ contributions

MCC designed and conducted experiments; wrote and edited the manuscript. GCR, SR, ISP and CF contributed to conducting experiments. ZA contributed to experimental design, wrote and edited the manuscript. All authors read and approved the final manuscript.
